# Cardiovascular risk management in patients with coronary heart disease in primary care: variation across countries and practices. An observational study based on quality indicators

**DOI:** 10.1186/1471-2296-13-96

**Published:** 2012-10-05

**Authors:** Jan van Lieshout, Richard Grol, Stephen Campbell, Hector Falcoff, Eva Frigola Capell, Mathias Glehr, Margalit Goldfracht, Esko Kumpusalo, Beat Künzi, Sabine Ludt, Davorina Petek, Veerle Vanderstighelen, Michel Wensing

**Affiliations:** 1 Scientific Institute for Quality of Health Care, Radboud University Nijmegen Medical Centre, PO Box 9101, 114, 6500 HB, Nijmegen, the Netherlands; 2 Health Sciences - Primary Care Research Group, (National Primary Care Research & Development Centre), University of Manchester, Williamson Building, Oxford Road, Manchester, M13 9PL, UK; 3 Université Paris Descartes, Faculté de Médecine, Département de Médecine Générale, 75015 Paris; Société de Formation Thérapeutique du Généraliste (SFTG), 233 bis rue de Tolbiac, 75013, Paris, France; 4 Instituto Universitario Avedis Donabedian (FAD), Universitat Autònoma de Barcelona, Provença 293 pral, 08037, Barcelona, Spain; 5 Department of Orthopedic Surgery, Medical University of Graz, Auenbruggerplatz 5-7, A-8036, Graz, Austria; 6 Clalit Health Services, 101 Arlozorov Street, P.O. Box 16250, Tel Aviv, Israel; 7 University of Eastern Finland, Department of Public Health and General Practice Kuopio Campus, P.O. Box 1627, FI-70211, Kuopio, Finland; 8 Swisspep Institut für Qualität und Forschung im Gesundheitswesen, Postgasse 17, CH-3011, Bern, Switzerland; 9 Department of General Practice and Health Services Research, University of Heidelberg, Voßstr 2, D-69115, Heidelberg, Germany; 10 Zdravje Medical Center, Smoletova 18, 1000, Ljubljana, Slovenia; 11 Domus Medica, Sint-Hubertusstraat 58, 2600, Berchem, Belgium

## Abstract

**Background:**

Primary care has an important role in cardiovascular risk management (CVRM) and a minimum size of scale of primary care practices may be needed for efficient delivery of CVRM . We examined CVRM in patients with coronary heart disease (CHD) in primary care and explored the impact of practice size.

**Methods:**

In an observational study in 8 countries we sampled CHD patients in primary care practices and collected data from electronic patient records. Practice samples were stratified according to practice size and urbanisation; patients were selected using coded diagnoses when available. CVRM was measured on the basis of internationally validated quality indicators. In the analyses practice size was defined in terms of number of patients registered of visiting the practice. We performed multilevel regression analyses controlling for patient age and sex.

**Results:**

We included 181 practices (63% of the number targeted). Two countries included a convenience sample of practices. Data from 2960 CHD patients were available. Some countries used methods supplemental to coded diagnoses or other inclusion methods introducing potential inclusion bias. We found substantial variation on all CVRM indicators across practices and countries. We computed aggregated practice scores as percentage of patients with a positive outcome. Rates of risk factor recording varied from 55% for physical activity as the mean practice score across all practices (sd 32%) to 94% (sd 10%) for blood pressure. Rates for reaching treatment targets for systolic blood pressure, diastolic blood pressure and LDL cholesterol were 46% (sd 21%), 86% (sd 12%) and 48% (sd 22%) respectively. Rates for providing recommended cholesterol lowering and antiplatelet drugs were around 80%, and 70% received influenza vaccination. Practice size was not associated to indicator scores with one exception: in Slovenia larger practices performed better. Variation was more related to differences between practices than between countries.

**Conclusions:**

CVRM measured by quality indicators showed wide variation within and between countries and possibly leaves room for improvement in all countries involved. Few associations of performance scores with practice size were found.

## Background

Many patients with chronic conditions are treated in primary care. This is challenging as high quality chronic care asks for an organizational structure allowing for population-based management. In previous research larger practice size tented to be related to higher quality of care considering various conditions with greater diversity of services [1-5]. Furthermore, larger practices tended to show more features consistent with the delivery of chronic care [6,7]. In many countries there is a tendency to develop larger practices [8]. Increasing size of scale and scope may be, up to a certain point, associated with decreasing average costs of a service as fixed costs like participation in continued education and hiring additional staff are divided by a larger number of patients. From an educational perspective, a larger size of scale with more patients may be associated with larger opportunity to practice specific procedures, thus steeper learning curves and higher quality of performance. On the other hand, a smaller practice size may have advantages in terms of more personal care and continuity [9].

Cardiovascular diseases (CVD) have major impact on the mortality and health-related quality of life of people in both developed and developing countries. Despite a declining cardiovascular mortality, improvements in the preventive, medical and surgical treatment in previous decades, and widely accepted practice guidelines [10-12], CVD are still one of the major causes of death and illness. Primary care can play an important role in delivering cardiovascular risk management (CVRM) to populations, but previous research showed that not all eligible patients receive optimal prevention of atherosclerosis-related CVD [13,14]. Many European countries therefore have adopted large scale programs for improving cardiovascular risk management, including pay-for-performance in the United Kingdom, disease management in Germany and practice accreditation in the Netherlands [15].

While data on CVRM are collected in a number of countries, mostly in specialized care settings [13,14], comparable data from primary care where many patients are treated and counselled, was lacking. We conducted an observational study of current CVRM in primary care in eight European countries, focused on patients with established coronary heart diseases (CHD) [16]. In this paper we aimed to describe current practice across countries and to explore associations of practice size with CVRM measured by quality indicators.

## Methods

Data were derived from the EPA Cardio study [16]. In this cross-sectional observational study eight countries provided data on CVRM in primary care practices: Austria, Belgium, England, France, Germany, Netherlands, Slovenia, and Switzerland. The country sample was a convenience sample from the countries participating within the EQuiP framework and included countries with a strong and with a weak position of primary care within the national health care system, and both small and large countries[17]. Countries with a strong primary care orientation were England, the Netherlands, and Slovenia; in the other countries primary care held a weaker position within the health care organization [15,18]. Data from patient records were gathered in 2008 and 2009. Ethical approval for the study has been obtained in each of the participating countries, according to national laws and regulations. A detailed study protocol has been published [16].

Stratified random sampling of 36 practices per country was planned, involving two factors: practice location (up to versus more than 100000 inhabitants) and practice size (up to two versus two or more full time equivalent physicians working in the practice). The relative contribution of each stratum should mirror the national situation and each country had the option to add strata in order to better reflect the national context. Four countries used this possibility: in England large practices were split in up to and more than five GPs; in the Netherlands small practices were split in single handed and duo; and in Germany and Slovenia the stratum up to 100000 inhabitants was split in up to and more than 40000 inhabitants. The number of practices – in relation to the patient numbers - was based on calculations of statistical accuracy, as described in the study protocol based on the detection of significant differences between indicator scores between two countries[16]. It was calculated that 36 practices per country with data on 15 patients per practice would suffice for this goal. Furthermore, earlier experiences with international comparative data showed that 30–40 practices can provide a reasonably good representation of the national situation [19].

### Patient population

We aimed at including 15 patients with established CHD per practice, including patients with myocardial infarction, angina pectoris or coronary interventions. Patients with diabetes were excluded because diabetes care and care for CHD patients are largely congruent. With diabetes patients included our results in part would be determined by diabetes care. In each practice a list of eligible patients was made, preferably based on coded diagnoses in the data files of patients registered with the practice. Then a random sample of 30 patients was taken from this list of CHD patients anticipating a 50% response rate. As variation across countries was anticipated related to the possibilities to generate patient lists, as second choice, other methods were allowed, for instance going through patient lists or simply by recalling patients. In Belgium, England, the Netherlands and Slovenia patient selection was exclusively based on recorded diagnoses. Apart from coded diagnoses, in Germany and Switzerland, the GP selected extra patients by recalling CHD patients in practices with less than 30 patients with a coded diagnosis of CHD. In two countries it appeared impossible to select patients based on coded diagnoses. In Austria, patient selection was based on going through prescription lists. In France, primary care physicians included eligible patients when they visited the practice. For this study we excluded practices with data on less than 8 CHD patients.

### Measures

Measures were linked to a set of rigorously developed performance indicators for CVRM [20]. To develop these indicators we used a RAND Modified Delphi procedure with two rounds of consensus, with 101 general practitioners from nine countries involved in the consensus process. From an original list of 650 indicators derived from the scientific literature, we first identified and edited 186 unique indicators. After two rounds of consensus 17 indicators relevant for patients with established CHD were selected; for 11 out of these 17 indicators data could be collected by extraction from medical records. These indicators comprised the registration of risk factors (smoking status, physical activity capacity, weight or body mass index, blood pressure, and serum cholesterol), advice on physical activity, influenza vaccination, antiplatelet and statin drug therapy prescribed or offered, systolic and diastolic blood pressure below threshold (140 and 90 mmHG respectively). Though not identified as a key-indicator we also present data on LDL-cholesterol levels because these data are widely seen as an important treatment goal [10-12]. All measures were systematically translated into the different countries’ languages, with established procedures of forward and backward translation and a pilot testing. The final instrument to collect the data from the patient records was tested and adapted in a pilot project in five countries including two practices [21].

### Analysis

We calculated descriptive figures per practice providing data on practice size and CVRM. For each practice the percentages of patients with a positive score on indicators were assessed; patients with a missing value were excluded for this outcome measure. We determined the mean scores across practices per country with standard deviations, implying that each practice had equal weight irrespective of the number of patients included. We tested whether the country means deviated significantly from the grand mean, using two-sided t-tests considering Levine’s test results. As the analyses of country differences are explorative a threshold value of p<0.01 was chosen to reduce the possibility of chance capitalization.

Based on the reported practice size - patient list size when available, otherwise yearly attending patient numbers - using a logistic multilevel regression analysis we assessed the association between indicator scores and practice size per country with two levels: a patient and a practice level. Age and gender were independent variables in the first level (patient level). In these analyses practice size was based on patient number as a continuous factor and not on the number of GPs in the practice what was used only for easy definition of stratification groups.

Furthermore, we performed a three level logistic regression analysis with country as a fixed factor in the third level. For this analysis we standardized practice size per country. With this methodology we corrected for the differences in practices sizes between the countries as we were not interested in country differences but in the effect of practices size across countries. Furthermore, this transformed patient numbers to comparable data in all countries, even comparing countries with numbers from patients lists and countries with numbers of attending patients. We assessed the association between practice size and indicator scores across all countries and across the countries with a strong and a weak primary care system apart. Related to practice size we hypothesized that larger practices would perform better; for these hypothesis driven analyses we used p<0.05 as threshold for significance.

We assessed the contribution of practices and of countries to the variance in scores on the performance indicators. The Intra Class Coefficients were computed based on the methodology described by Twisk [22].

SPSS 16 was used for descriptive analysis and t-tests, SAS for random coefficient regression modelling.

## Results

In several countries it appeared impossible to include 36 practices. Finally 232 practices participated (81% of the number aimed at). We excluded 51 practices because they did not provide data on practice size (n=14), included less than eight CHD patients (n=33), or both (n=4). In this study we included 181 practices. In Austria and Switzerland a convenience sample of practices participated; in Belgium additional to practices from a list four practices were included after they were personally contacted by the researchers. All other countries worked with national or regional practice lists. Practices in Austria, Germany, and Switzerland reported on the yearly attending population; in the other countries practice size was based on the number of patients listed.

The 181 practices included provided data on 2960 patients, on average 16.4 per practice. Overall 33% of the patients included were female and the overall mean age was 68.7 years (see Table 1).

**Table 1:** Practice sample and demographic data.

	**Practices (n)**	**Mean practice size (sd)**	**Patients (n)**	**% female**	**Mean age (years)**
Austria	23	2878^*^ (1369)	293	36	71.6
Belgium	18	3035 (2363)	232	25	66.7
England	32	6573 (3655)	479	39	68.2
France	9	1417 (754)	133	26	67.8
Germany	13	4423^*^ (1608)	248	35	70.0
The Netherlands	34	3183 (1215)	495	29	69.3
Slovenia	35	2059 (804)	805	36	68.3
Switzerland	17	3449^*^ (2537)	275	24	68.2
total	181	3538 (2582)	2960	33	68.7

^*^ Practices size provided by practices as number of yearly attending patients; in all other countries as number of patients listed.

### Cardiovascular risk management

Regarding cardiovascular risk factor recording, the percentage of missing values was consistently 3 to 4%. The mean practice score of recording physical activity capacity was, on average, about 50% (see Table 2). Overall, blood pressure recording had the highest score (94%), followed by cholesterol levels (87%). Standard deviations are indicative of the differences between practices.

**Table 2:** Indicator scores: risk factor recording

		**Smoking status**	**Physical activity capacity**	**Weight/BMI**	**Blood pressure**	**Serum cholesterol**
Austria	Mean	100↑	50.6	61.4	94.2	95.7↑
	SD	0	31.2	33.0	7.1	6.0
	p	.000	.566	.311	.957	.000
Belgium	Mean	76.7	52.6	84.4↑	98.0↑	95.4↑
	SD	29.0	34.5	15.1	4.2	6.7
	p	.708	.805	.001	.004	.000
England	Mean	94.8↑	64.1	82.9↑	98.3↑	94.6↑
	SD	10.0	29.4	22.1	3.4	7.5
	p	.000	.121	.003	.000	.000
France	Mean	79.4	46.0	90.9↑	96.2	96.4↑
	SD	21.5	39.6	11.0	7.7	6.7
	p	.983	.439	.000	.555	.002
Germany	Mean	92.5↑	55.6	65.2	96.3	94.2
	SD	9.9	41.5	33.3	7.0	8.9
	p	.001	.933	.662	.462	.016
Netherlands	Mean	57.2↓	41.7	44.6↓	82.0↓	67.2↓
	SD	28.3	27.7	27.3	15.8	19.5
	p	.000	.030	.000	.000	.000
Slovenia	Mean	76.5	60.0	70.8	97.4	83.6
	SD	26.2	29.0	25.0	67	14.4
	p	.580	.358	.705	.021	.253
Switzerland	Mean	65.4	62.8	71.4	96.6	87.8
	SD	38.1	34.8	24.4	5.9	10.8
	p	.162	.323	.727	.324	.830
Total	Mean	79.3	54.6	68.8	94.1	86.9
	SD	27.5	32.2	28.8	10.4	16.0

Mean practice scores (%) per country and across countries with standardized variation. Scores significantly deviating from the mean of all countries are marked when p<0.01, with p values displayed.

Indicators concerning achievement of target values for SBP, DBP and LDL, considering the most recent measurements, are displayed in Table 3. We had data of about 90% of the patients. Overall, the mean practice score on the indicator DBP below 90 mmHg was 85%, and on SBP and LDL cholesterol about 45%. On average the scores on the recommended cholesterol lowering and anti-platelet drug treatment were 80%. The practice mean score on influenza vaccination was less than 70%.

**Table 3:** Indicator scores

		**Advice/contraindication physical activity**	**Influenza vaccination**	**Antiplatelet therapy if not contra-indicated**	**Statin recorded/offered**	**SBP below threshold**^**1**^	**DBP below threshold**^**2**^	**LDL below threshold**^**3**^
Austria	Mean	61.1	49.9	86.4	78.8	46.7	86.0	59.1
	SD	30.8	29.2	25.1	22.5	20.9	9.5	22.5
	p	.062	.012	.739	.317	.850	.871	.022
Belgium	Mean	39.3	90.7↑	90.1	85.8	55.4	85.0	46.6
	SD	32.1	12.2	12.3	12.2	21.3	12.6	16.3
	p	.317	.000	.553	.427	.065	.870	.816
England	Mean	48.1	87.3↑	90.8	90.2↑	43.3	95.7↑	65.5↑
	SD	30.7	14.4	10.5	10.8	14.8	5.1	15.1
	p	.917	.000	.171	.002	.404	.000	.000
France	Mean	45.7	50.7	88.0	86.4	61.4	90.2	38.9
	SD	39.0	31.9	15.3	21.4	18.3	10.7	19.6
	p	.876	.114	.966	.513	.029	.260	.247
Germany	Mean	56.3	74.9	69.3	69.5↓	52.3	81.2	37.1
	SD	45.0	31.4	25.4	17.1	20.5	10.8	13.8
	p	.497	.520	.023	.006	.282	.204	.090
Netherlands	Mean	28.8↓	96.8↑	82.8	77.9	28.9↓	81.0	43.0
	SD	22.9	5.8	18.2	15.9	15.6	12.7	20.8
	p	.000	.000	.129	.120	.000	.046	.261
Slovenia	Mean	54.9	33.3↓	92.9↑	84.2	46.0	80.2	37.8
	SD	29.5	32.6	8.9	13.2	18.7	12.5	20.7
	p	.212	.000	.010	.612	.968	.018	.016
Switzerland	Mean	52.3	56.9	94.1	84.7	59.8↑	87.3	41.5
	SD	38.9	28.1	9.9	15.2	23.4	11.5	23.7
	p	.567	.159	.128	.625	.010	.570	.303
Total	Mean	47.4	68.8	87.7	82.7	45.8	85.5	47.5
	SD	33.0	33.5	16.9	16.3	20.9	12.1	21.8

Mean practice scores (%) per country and across countries with standardized variation. Scores significantly deviating from the mean of all countries are marked when p<0.01, with p values displayed.

^1^ SBP below threshold: SBP<140 mmHg.

^2^ DBP below threshold: DBP<90 mmHg.

^3^ LDL below threshold: LDL<2.5 mmol/l.

Risk factor recording in general was below the mean in the Netherlands; in England most factors were recorded significantly more often. The other countries showed less deviations from the grand mean scores (see Table 2). Considering the outcomes advice on physical activity, influenza vaccination, antiplatelet and statin drug therapy, blood pressure and cholesterol levels (see Table 3) England again scored above the mean in 4 of the 7 outcomes. Here the Netherlands and Slovenia outperformed on one outcome, respectively influenza vaccination and antiplatelet drug therapy.

### Practice size

In the analyses per country practice size did not consistently correlate to the outcomes (data not shown). In Slovenia 4 of the 12 outcomes (recording of physical activity capacity and BMI or weight, advice or contraindication for physical activity, influenza vaccination) had a significant positive association with practice size; 7 outcomes were non-significant positive and one was non-significant negative. We found one other significant association: In England practice size and the indicator score related to influenza vaccination were negatively associated.

For the outcome measure recording of physical activity capacity all countries showed a positive association, though significant only in Slovenia.

Across all 8 countries we found no association between practice size and indicator scores. In countries with a strong primary care system practice size was positively associated with the score on influenza vaccination (OR 1.28, 95%CI 1.01 – 1.61) and negatively with the LDL cholesterol level score (OR 0.86, 95%CI 0.74 – 0.99). In countries with a weak primary care system we could not detect associations between practise size and outcomes.

We assessed the relative contribution of practices and countries to the variance in indicator scores, the ICC scores (See Table 4). Of the indicator scores on SBP, DBP and LDL about 10% of the variance could be explained at the practice and country level together. In all other indicators more of the variance could be explained, about 15-30% at the practice level and up to 18% at the country level.

**Table 4:** Intra Class Coefficients

**Indicator**	**Country level**	**Practice level**
Smoke status recorded	11.6	31.7
Physical activity capacity recorded	0.9	29.1
Weight or BMI recorded	7.6	24.7
Blood pressure recorded	16.4	28.4
Cholesterol recorded	17.0	19.3
Advice or contraindication for physical activity	2.1	28.6
Influenza vaccination	18.2	17.9
Antiplatelet therapy	6.1	26.1
Statin advised or prescribed	2.5	15.2
SBP below threshold	3.4	7.9
DBP below threshold	3.8	7.4
LDL below threshold	3.6	6.5

## Discussion

This is, to our knowledge, the first study on CVRM in European primary care at a larger scale. We found that scores on quality indicators in general vary from 45% (a record of advice on physical activity; SBP and LDL below treatment targets) up to about 95% (blood pressure recording) of the maximum score, which indicates optimal policy. As opposed to our expectation, we found little evidence for better performance in large practices. In Slovenia larger practices tended to perform better. Our study did not explicitly assess the efficiency of delivering CVRM.

Similar to our research, the three EUROASPIRE surveys provide data from international research with uniform data collection across countries. But in EUROASPIRE a specialist care starting point guided CHD patient selection [23]. Raised blood pressure, defined as SBP≥140 mmHg and/or DBP≥90 mmHg or in diabetics respectively ≥130 mmHg and ≥80 mmHg, was prevalent in 58-61% of these survey samples. Raised cholesterol was defined as ≥4.5 mmol/l and diminished from 94.5% in EUROASPIRE I, to 76.7% in the second survey and finally to 46.2% in the third. Data collected in the most recent EUROASPIRE survey in 2006 and 2007 are comparable to our results. In the Pinnacle programme, data regarding outpatients from cardiology offices, too, show comparable results with for instance antiplatelet and statin therapy in 84.9% and 84.3%, respectively [14]. Previous data on CVRM in primary care can be found in various national studies. In a Cochrane review the effects of interventions on the organisation of the treatment considering ischemic heart disease patients in primary care are studied. [24]. Data from the control groups could be considered comparable to our audit data. Direct comparable outcomes are statin prescription and antiplatelet therapy. In the review 50.1 of the control patients received statin therapy, but studies dated back till the 1990’s. The most recent study, SPHERE, had with 80.3% a result comparable to 82.7% in our study. Relating to antiplatelet therapy the review result was 72.5% compared to 87.7 in our study sample. Again, the more recent data were the best, up to 87.0 In the SHERE study SBP was <140 mmHg in 66.2% (versus 47.5% in our data) and DBP<90 mmHg in 88.6% of the patients (comparable to 85.5% in our data).[25] In drug trials efficacy of statins varies from 60 to 90% in achieving LDL < 2.5 mmol/l [26-29]. In our observational study, the real life results are on the lower end of this range. In contrast to the optimum situation in these drug studies physicians could include every patient known to have a CHD, patients without further medical attention, too. The indicator on LDL cholesterol treatment target surprisingly was not validated in the Delphi indicator development procedure. We can only speculate about the reasons; setting strict norms irrespective of the patient’s age might be argued by some or the fact that this outcome measure very much depends on the patient in contrast to process measures as offering a statin. In view of the strong evidence base for the relationship between LDL cholesterol and coronary heart disease we anyhow decided to include the LDL cholesterol results in our study.

Since most patients with increased cardiovascular risk are treated in primary care, the findings are relevant for improving care in the different countries despite study limitations. They show that specific countries scored high on some indicators and low on others. Improvements in CVRM are possible in all countries. Our study allowed to include all patients with a known diagnosis of CHD. Inevitably, patients treated in secondary care could be included, too. Our results give an overview of the performance of CVRM related to all patients known in the primary care practice.

In England high scores on performance indicators were observed, particularly for indicators incentivized as part of the Quality and Outcomes Framework (QOF) [30]. Physicians in England are forced, by their electronic patient records, to tick boxes for QOF-indicators, which might be a strong driver for change in registration, enhancing good risk factor registration in England. On the other hand, we found relatively low performance scores for some indicators of CVRM, especially risk factor recording, in the Netherlands. The only indicator related to a financial incentive (influenza vaccination) and supported by a national organizational programme had very high scores in this country (in 2009 a fee of 9.88 euro was provided for every vaccinated patient). The system parameter incentives on a national level and as such as a country characteristic may have an important influence relative to practice size as a practice characteristic.

The DBP indicator scores were much higher than scores for SBP, though the importance of the latter is stressed by its role in risk classification schemes. Advice on physical activity had low scores, too, although it remains uncertain whether such advice had been provided but not recorded.

Differences between countries may be partly explained by differences in the quality of recording as stated above. Medication and blood pressure or cholesterol levels are probably well recorded, but this is less the case for smoking status or exercise advice. It might be argued that recorded care does not mirror care provided. But in chronic care recording is thought to be essential. Risk factor recording is a prerequisite to select patients for treatment and chronic care means collaboration between various health care professionals, who will need to rely on the data in the patient records [31].

In our study practice size seems to have little relation to performance as measured by quality indicators. Though in previous research on practice size no consistent results were found, in general larger practices tend to show better performances and provide more extensive services, for instance more preventive activities [1-5]. All these studies were based on national data. We took into account the fact that we had practices from eight countries by entering country as a level in our multilevel analysis. This procedure effected chance on significant findings. Taking into account the strength of the primary care did not provide relevant findings.

A larger practice offers opportunities to develop skills by experience and gives managerial advantages, especially when specialized staff is required. Structured care will be more cost effective with larger patient groups included in a program. On the other hand, there seems to be a trade of between high quality clinical care and interpersonal care, and access might be better in smaller practices [2,9]. In our sample across countries small practices were able to deliver a performance on cardiovascular risk management as good as larger practices.

Only in Slovenia larger practices showed a tendency towards better performance in general. We can only hypothize on this finding. It may be the resultant of recent implementation strategies with first effects in larger settings. This would be in line with the general concept of larger practices being in a good position for providing structured care to larger groups of patients.

The proportion of variance explained at the practice level was larger than that related to the country level, indicating that the practice has more influence on that variation than the country. This could stimulate practices to invest in quality improvement in their practices as there is little argument that much is determined at a higher level out of their reach. A remarkable small part of the variation in outcomes is explained at both the practice and the country level considering the blood pressure and cholesterol levels. These biological outcomes will be determined at the patient level to a greater extend.

### Strengths and limitations of the study

Within the context of our international survey we had to face inclusion bias both at the practice level and at the patient level as a result of differences in the organization of the health care system within the various countries at different levels. Practice selection was random in most countries but a convenience sample in two countries (Austria and Switzerland). The procedure for sampling patients, too, showed some variation. In Belgium, England, the Netherlands and Slovenia patient selection was exclusively based on recorded diagnoses, enabling inclusion of patients registered but not controlled in primary care or not at all. Less strict methods were used in the other countries (remembering patients, prescription lists, attending patients) providing patient inclusion bias. Patients on a prescription list by definition have some drug treatment and frequent attenders and treated patients are more likely to be remembered. Our practice sample appeared the best feasible given the limitations of our international survey. The sample size of 181 practices forms a limitation to detect small effects of practice size on outcomes, among other due to clustering within countries and differences of possible effects between countries.

Data on practice size were not directly comparable between countries because of the differences in health care systems. In some but not all countries patients are listed with one GP or practice. Countries without these clear patients’ lists had to report on numbers of attenders as a measure for practices size. By standardizing practice size data per country we solved this potential problem.

We included patients with CHD to have a patient group more homogeneous than the group of CVD patients in general. This did not completely prevent heterogeneity within our study population. The CHD group comprised on the one hand patients who had a myocardial infarction or vascular surgery and have been treated in secondary care and on the other hand patients with stable angina pectoris who might have been treated in primary care exclusively.

### Conclusions

The variation between practices within each country is unwanted and proves potential for improvement. The presence of highly performing practices within each country proves that in each national context good CVRM is possible. Differences found between countries and especially best practices can form lessons for all countries. For instance the Quality and Outcomes Framework from the UK can be an example to other countries but focus may differ according to the national situation as the position of primary care within the larger context of the health care system.

In contrast to most previous research our analysis did not indicate significant influence of practice size on the quality indicator scores. In various studies larger practices tend to perform better, supporting the development of practice collaboration with consequently larger groups of CHD patients to organize care. This may enhance expertise and logistics. We could not confirm this tendency. Here, further research is needed.

## Competing interests

The authors declare that they have no competing interests.

## Authors' contributions

MW and RG designed the Epa Cardio project to which all authors contributed in the project meetings. All authors participated in data acquisition. JvL and MW led the analyses and interpretation of the data. MW, RG and JvL drafted the manuscript. All authors commented the drafts; JvL integrated the comments. Finally, all authors read and approved the final manuscript.

## Pre-publication history

The pre-publication history for this paper can be accessed here:

http://www.biomedcentral.com/1471-2296/13/96/prepub
